# Structural Topic Model Analysis of Mask-Wearing Issue Using International News Big Data

**DOI:** 10.3390/ijerph18126432

**Published:** 2021-06-14

**Authors:** Kyeo Re Lee, Byungjun Kim, Dongyan Nan, Jang Hyun Kim

**Affiliations:** 1 Department of Human-Artificial Intelligence Interaction, Sungkyunkwan University, Seoul 03063, Korea; happyeffect@gmail.com (K.R.L.); ndyzxy0926@skku.edu (D.N.); 2 Department of Interaction Science, Sungkyunkwan University, Seoul 03063, Korea; kuntakim88@gmail.com

**Keywords:** structural topic model, International newspaper, quarantine, mask-wearing, COVID-19

## Abstract

Media plays an important role in the acquisition of health information worldwide. This was particularly evident in the face of the COVID-19 epidemic. Relatedly, it is practical and desirable for people to wear masks for health, fashion, and religious regions. However, depending on cultural differences, people naturally accept wearing a mask, or they look upon it negatively. In 2020, the COVID-19 pandemic led to widespread mask-wearing mandates worldwide. In the case of COVID-19, wearing a mask is strongly recommended, so by analyzing the news data before and after the spread of the epidemic, it is possible to see how the direction of crisis management is being structured. In particular, by utilizing big data analysis of international news data, discourses around the world can be analyzed more deeply. This study collected and analyzed 58,061 international news items related to mask-wearing from 1 January 2019 to 31 December 2020. The collected dataset was compared before and after the World Health Organization’s pandemic declaration by applying structural topic model analysis. The results revealed that prior to the declaration, issues related to the COVID-19 outbreak were emphasized, but afterward, issues related to movement restrictions, quarantine management, and local economic impacts emerged.

## 1. Introduction

The coronavirus strain of SARS-CoV-2 (COVID-19) became an epidemic in late 2019, transitioning into a World Health Organization (WHO) pandemic declaration in March 2020. Spanish Flu (H1N1 influenza A virus) was a worldwide pandemic, spreading from 1918 to 1920 prior to coming under the annual “flu-shot” mitigation controls that persist to this day. Notably, the worldwide and ubiquitous aviation industry accelerated the spread of Spanish Flu and coronavirus. As with any public emergency, the media plays an important role as both a spreader of useful health information as well as anxiety-related behaviors. Citizens obtain information about diseases through the media and decide how to deal with them based on their personal, familial, cultural, political, and religious values. The role of the media is important in obtaining information about the spread of the virus [1]. It is therefore important to analyze the COVID-19-related media information before and after WHO’s declaration to better understand the worldwide social attributes at play. Since March 2020, the mask-wearing theme has proliferated cultural discourse worldwide. Thus, analyzing this theme through the lens of the media is relevant to understanding their role as a functional mediator. We set a research period for data collection from 1 January 2019 to 31 December 2020 to differentiate differences in perception related to mask-wearing before and after the pandemic declaration. The data collection period was constructed to begin about 1 year before the epidemic of COVID-19 until the pandemic had spread worldwide. We analyzed the content of media reporting before and after the pandemic declaration based on international news articles to understand the public, theoretical, and practical implications.

## 2. Literature Review and Theoretical Elaboration

### 2.1. Crisis Risk Management and News Media

Since the 2001 9/11 terrorist attack in New York City, the importance of crisis management has increased significantly. This is because terrorism threatens the physical and mental well-being of a large number of people, owing to the terrifying incidents, the numerous loss of life, and the economic impacts [2,3]. Crises, by definition, are sudden and destructive; they destabilize communities and nations and thus require immediate recovery actions. The term “crisis” is utilized to describe many incidents, including disease outbreaks. In such an environment, the utility of the media is highly utilized as a communication channel directly from agencies to citizens. Hence, the media plays a direct role in protecting society and citizens. Furthermore, a crisis is a sudden and destructive event that destabilizes all normal activities of the community and requires immediate action for recovery. Such a crisis includes large-scale earthquakes, damage from floods and droughts, and large-scale blackouts. These discussions further define an infectious disease that has not been experienced as a crisis. In this environment, the need for media is emphasized as a communication channel on how to recover from disasters and reduce damage. The media plays a role in protecting citizens by delivering timely messages.

Several studies [4,5] have implied that providing citizens with accurate information related to certain crises is key to overcoming them. Several scholars have indicated that effective crisis communications improve communities’ ability to resist and overcome adversities [6,7,8]. With this viewpoint, many governments have attempted to provide accurate and timely information about crises through news media channels when available. Furthermore, disasters not only included warning alerts but also aided in situational awareness. Song et al. [9] explored the spreading patterns of fake information about MERS by analyzing 8,671,695 related online documents in Korean online channels. Yang and Lee [5] explored how information crises affected the mainstream media through content analysis of online news channels during the Korean MERS outbreak. From the analysis of online news in English over 5 years, Lansdall-Welfare et al. [10] found that the attitude and sentiment of the media toward nuclear energy in the Fukushima disaster changed significantly.

Such studies imply that pattern exploration and sentiment analysis from online news sources contribute to guiding society to overcome crises. Thus, we feel that analyzing news data from the COVID-19 pandemic can lead to better crisis management.

### 2.2. COVID-19 Crisis Management

During the COVID-19 pandemic, worldwide media has widely reported pandemic-related stories. Thus, the news media have become an effective source of conveying social, economic, and political realities in response to the deadly consequences of this virus. Additionally, the WHO’s risk communication team established an information portal (i.e., the WHO Epidemic Information Network) to offer effective and evidence-based information about COVID-19 [11]. For a better understanding of the patterns of COVID-19-related news, several scholars have analyzed news data in terms of contributing to and managing the crisis.

Park et al. [12] used the modeling results of tweets about COVID-19 from four Asian countries (i.e., Korea, Vietnam, etc.) to explore similarities and differences of pandemic-related social-media discourse. Kaveh–Yazdy and Zarifzadeh [13] found that the main concerns of the Iranian public included topics related to “PCR lab, test, diagnosis, and screening”, “closure of the education system”, and “awareness actions about washing hands and facial mask usage”, based on the latent Dirichlet allocation (LDA) topic modeling results of 2,400,000 posts from official news channels. Liu et al. [14] conducted another LDA topic model to analyze 7791 Chinese news articles about COVID-19 and discovered that prevention, control procedures, medical treatments, research, and global or local social and economic influences were the key themes.

According to the literature related to news media coverage of COVID-19, we found that existing studies rarely focused on exploring the patterns of news articles in multiple countries across different cultures. Therefore, this study examines news articles from 16 countries to fill the gap.

### 2.3. Computational Approach for Social-Issue Analysis

Various analytical methods have been applied to analyze social issues from the perspective of communication studies, depending on the purpose of the study. Traditional analysis methods, such as questionnaire surveys, human coding analyses, and qualitative studies, are a few examples. Recent social-science studies have used computational methods, such as applied semantic network analysis, topic-modeling analysis, and machine-learning evaluations to uncover more subtle causes and effects.

In particular, the application of computational analysis methods has a great advantage because it allows for the analysis of a large number of data [15,16]. Lee [15] argued that news-frame analyses, which are conducted based on human coding, can be implemented by topic modeling and network analyses based on cultural and societal issues. For this reason, Kim [17] applied topic-modeling techniques to provide a content analysis of news during the spread of COVID-19 using Korean news data. Furthermore, health information was analyzed via topic modeling based on news reports and Twitter content [18].

### 2.4. Mask-Wearing and COVID-19

Wearing a mask in public places is a simple, clear, and effective method of controlling the spread of infectious diseases. Additionally, masks are commonly worn by people of different cultures to express religious beliefs or cultural distinctions. During the COVID-19 pandemic, mask-wearing was directed by many countries, and doing so had a significant effect on the mitigation and elimination of COVID-19 deaths. Nevertheless, some individuals consider mask-wearing to be a negative practice of submission; thus, they resisted it. Many individuals believe that mask-wearing violates their personal freedoms of expression. Under these circumstances, several mass-media outlets have provided mask-wearing information to address several cultural dimensions. However, few scholars have investigated the specific public perspectives of wearing a mask [19,20]. Rieger [19] discovered that the perceived risk of COVID-19 and the aversion to mask-wearing played significant roles in individual behaviors. Furthermore, because mask-wearing has, in many cases, been forced or otherwise strongly recommended in certain locales, objections unrelated to health care have emerged. Our objective is to examine how these perspectives are unfolding based on international news reporting.

Overall, we intended to analyze the news-media topics reported internationally between 1 January 2019 to 31 December 2020 to examine the various opinions and responses as revealed from the media. From this, mask-wearing sentiment can be tracked over time. This leads us to two key research questions: Research Questions:

1. What are the key topics of the international news about mask-wearing based on before and after the WHO pandemic declaration?
2. How are these topics interlinked (proportion vs. correlation)?

## 3. Materials and Research Model

### 3.1. Data Crawling

**Figure 1:**
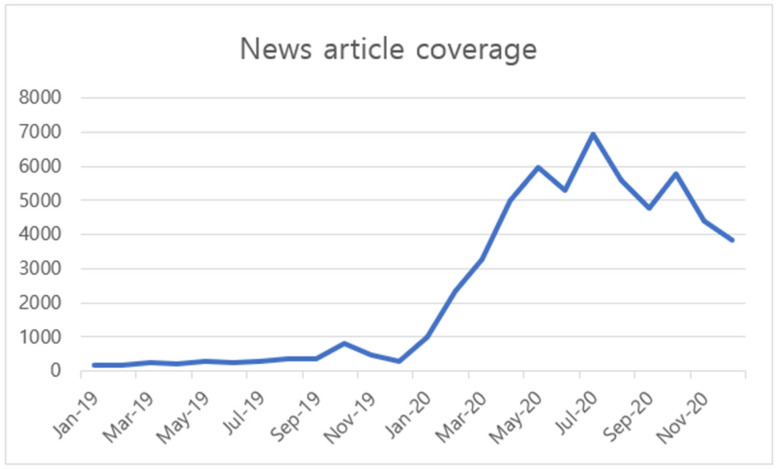
Count: news-article coverage, 2019–2020.

If news from multiple countries with various categories of news data is collected and analyzed within research that uses news as an analysis target, it can be called analysis using big data. We obtained news-media data about mask-wearing from LexisNexis (http://www.lexisnexis.com, accessed on 21 April 2021). We collected news data from major world newspapers and newspapers from Korea, China, Japan, Germany, and France. We analyzed the given topic of mask-wearing before and after the COVID-19 pandemic declaration from 2019 and 2020. With the spread of COVID-19, the volume of news articles showed an increasing trend. Monthly article report volumes are shown in Figure 1. Looking at the trend of news coverage, news reports have risen sharply since early 2020. This change in news coverage indicates that the COVID-19 issue was emerging as an important issue internationally.

### 3.2. Preprocessing

We preprocessed the data using Python 3.8.4 (https://www.python.org/, accessed on 21 April 2021), and refined the data using pandas, numpy, genism, and flashtext in the Python environment, performing natural language processing with the nltk package. In the python environment, we deleted unnecessary columns and set time-series data as variables for analysis. We also deleted duplicate data. After preprocessing, the remaining data available for analysis included 58,061 articles. We combined the content of news headlines and their constituent article texts, which are reflected in the contents column of Table 1. These data were converted into token units using Python’s morpheme analysis package. When two words appearing simultaneously in the conversion process exceeded a certain number of iterations, they were treated as compound words (e.g., “white_house”). Data preprocessing was completed by deleting stop words and unifying synonyms. After processing, data were further refined using stm (https://cran.r-project.org/web/packages/stm/vignettes/stmVignette.pdf, accessed on 21 April 2021) package in R4.0.3. Thus, we deleted duplicate articles having similarities greater than 0.9 using the “LexisnexisTools” (https://cran.r-project.org/web/packages/LexisNexisTools/index.html, accessed on 21 April 2021) package. Only articles that always contained the word “mask” were set as an analysis target.

### 3.3. Data Labeling

To facilitate data analysis, the preprocessed data were structured into data frames. Prior to labeling, we selected articles from media sources that reported at least 10 news items.

After confirming the location of each media company’s headquarters and their respective issuing country, labeling was conducted by dividing the companies into regions. We labeled cluster information corresponding to the country data of each company. The continental region information was divided into “Asia”, “Europe”, and “North America” in the cluster column. “Australia”, “Israel”, and “North Africa” sources were assigned to the “Europe” region. Cluster grouping was formed from live discussions among researchers based on national information about the major world newspapers from LexisNexis.

Articles from a total of 100 international media companies were then used for data analysis. Table 1 summarizes the top-20 media companies in order of the number of articles, revealing the numbers of news articles and regional cluster information.

### 3.4. Modeling

We analyzed the refined text data using a structural topic model (STM), which provides various topic analysis methods through which sophisticated text analysis was achieved [21]. Topic modeling uses Bayesian estimation to search for various topics in a large document set, assuming that various topics are latent in a set of individual documents. Using this analysis method, potential topics can be retrieved using the word distribution of all documents in the corpus [22,23]. STM is a natural-language processing algorithm that finds key topics in a set of documents; unlike other topic-modeling methods, it analyzes the correlations among topics. Because STM can check residuals for the number of topics, the validity of the model can be evaluated. Table 2 shows the parameter index used for STM analysis.

## 4. Results

**RQ1: What are the key topics in the international news about mask-wearing based on before and after the WHO pandemic declaration?**

On 11 March 2020, the WHO issued a pandemic declaration about COVID-19, recommending an international effort to stem the devastation. Afterward, most of the emphasis was placed on basic hygiene management and mask-wearing. To analyze the media responses to these international actions, we conducted a topic analysis on mask-wearing before and after the pandemic declaration.

**Figure 2:**
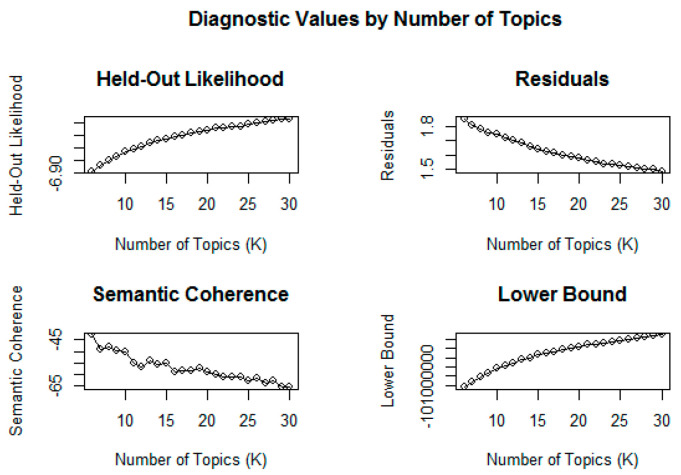
Diagnostic values by the number of topics.

First, we estimated the optimal number of topics from the refined media article data. Six to thirty topics were estimated, and the results are shown in Figure 2. The higher the values of the hold-out likelihood, the lower bound, and the semantic coherence, the higher the meaning of the topic. Furthermore, the lower the value of the residuals, the higher the density of meaning.

In Figure 2, the value of hold-out likelihood and lower bound is shown to have increased linearly with the number of topics. Meanwhile, the value of residuals steadily decreased as the number of topics increased. The value of semantic coherence decreased or increased as the number of topics increased. Thus, we performed STM analysis by setting the optimal number of topics to 13 based on this index.

STM topic analysis revealed a variety of high-level word types. Among these, the most commonly used method was the frequency and exclusivity (FREX) model. From this, it was easy to identify words having rare and important meanings among a set of words inherent to a topic. Table 3 shows 13 news-topic terms related to mask-wearing between 2019 and 2020.

**Figure 3:**
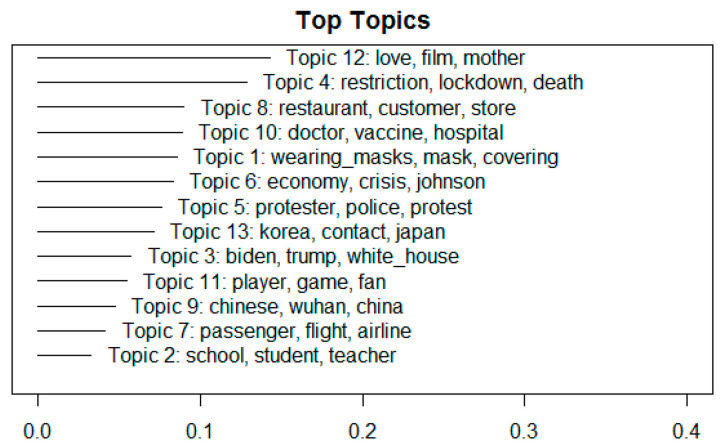
Expected topic proportions.

Figure 3 presents a graphical representation of the indicators for expected topic proportions. It shows that Topic 12 captures the largest proportion of all items, and Topic 2 captures the smallest proportion.

Table 4 shows the statistical estimation effect. When comparing topics before and after the pandemic declaration, topics having statistically significant differences are T5, T6, T7, T9, and T13. The T5—Protesting topic appears to more predominant after the WHO pandemic declaration. The T6—Economic topic is also located close to the period after the declaration. However, T7—Aviation industry, T9—Outbreak of COVID-19 in China, and T13—Quarantine management topics were found to be used before the WHO pandemic declaration.

**Figure 4:**
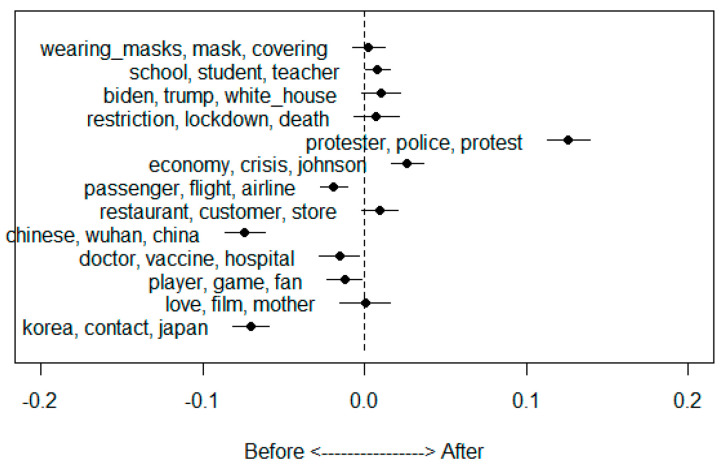
The estimated effect on topics before and after the WHO pandemic declaration.

Figure 4 represents the estimated effect discussed above. In sequence, the top point is Topic 1, and the last point is Topic 13. The displayed words are the top words from the FREX indicator.

**Figure 5:**
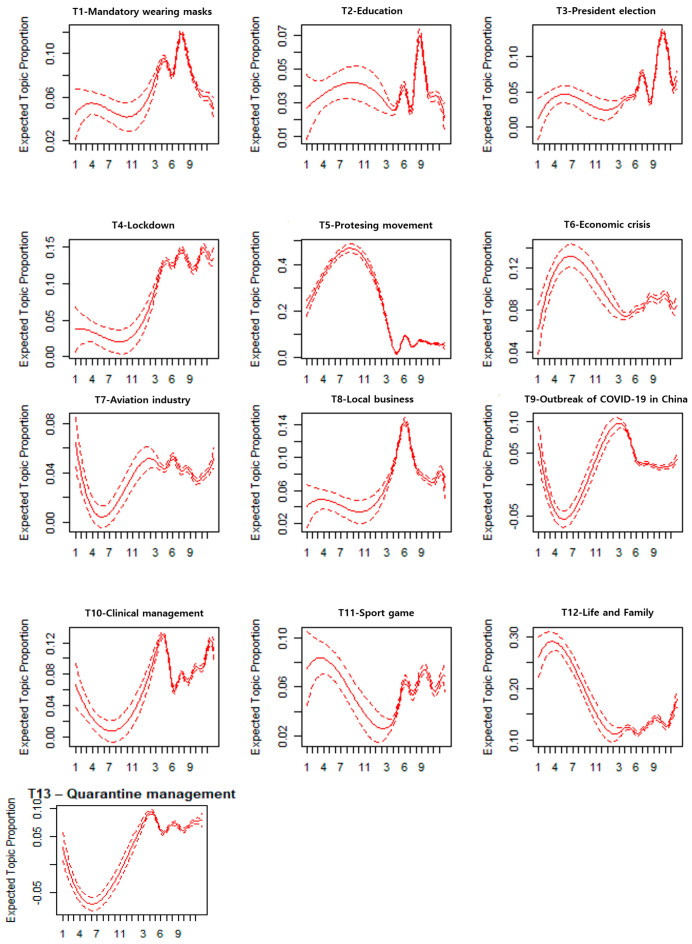
Expected topic proportion.

Figure 5 displays the changes in topic weight over time. In the case of Topic 1, the proportion of topics gradually increased after the pandemic declaration. This is coincident with the global spread of COVID-19. In the case of Topic 2, because non-face-to-face education was recommended, a trend of changing education methods and related issues were shown to increase, as emphasized at the beginning of the period. In the case of Topic 3, the political issue of mask-wearing in the US was related to their presidential election. For Topic 4, the issue of movement restrictions increased as COVID-19 spread internationally. In Topic 5, a resistance movement against mask-wearing mounted, and the expected topic proportion (ETP) increased in 2020. For Topic 6, topics related to the international economic crisis related to COVID-19 were emphasized. In Topic 7, after the pandemic announcement, the aviation industry topic emerged. For Topic 8, ETP increased rapidly in 2020, owing to local economic crises. For Topic 9, the graph is similar to that of Topic 8. Topic 10 indicates that health management issues were increasing rapidly. Topic 11 emphasized the temporary suspension of sports competitions and the conversion to non-audience expositions. In the case of Topic 12, mask-wearing themes in daily life and at home appear. Topic 13 reflected quarantine measures in Korea, Japan, and other countries.

**RQ2: How are these topics interlinked (proportion vs. correlation)?**

**Figure 6:**
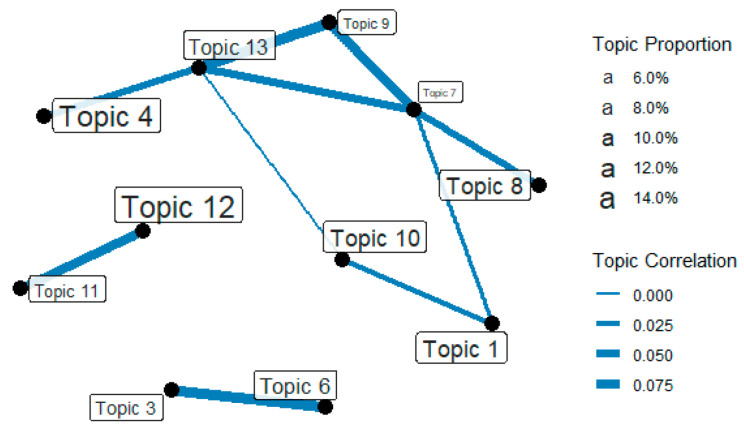
Topic network.

Figure 6 displays the network of topics that indicate the degree of relevance among topics. The larger the font size, the higher the weight; the thicker the line connecting the topics, the higher the correlation. This analysis was performed so that topics without connectivity could be deleted.

The aviation industry (T7) and quarantine management (T13) topics existed on the central axis of the greatest network connection, and topics related to moving restrictions (T4), clinical management (T10), and local businesses (T8) were high in proportion. This shows that international movement was restricted, and regional economies were severely affected by movement restrictions. However, sports (T11) and life and family (T12) events were highly interconnected, which seems to have increased interest in non-audience sports, owing to movement restrictions. Additionally, the economic crisis (T6) and the US presidential election (T3) were linked, suggesting that the combination of COVID-19 quarantines and the economic crisis affected the presidential election.

Analysis of the topic network structure showed that the quarantine management topic was critical to the topic network. To interpret meanings, the aviation industry and quarantine management played important roles in the spread and containment of COVID-19. Hence, the problems of mask-wearing reflected the connections among medical recommendations and regional economic policies under the larger theme of quarantine management. From the international perspective, aviation industry-related topics occupied an important position in the network because air travel was one of the most important factors contributing to the spread of infectious diseases across borders.

## 5. Discussion

This study analyzed the content of news reports regarding mask-wearing as reported in the international media from 1 January 2019 to 31 December 2020. This research provides an exploratory report on an STM algorithm that determines topics related to pandemics and quarantine that were latent in a large number of news articles. From the consideration of the hold-out likelihood, semantic coherence, residuals, and lower bounds found 13 news articles as optimal out of 58,061 cases.

The extracted topics clearly showed various issues regarding the COVID-19 pandemic. Furthermore, mask-wearing was found to have been a key action related to prevention. This study revealed through topic analysis that this pattern had a direct influence on the private lives and external activities of citizens worldwide.

Regarding cases of life and family (T12) topics, the topic weight was the highest. Thus, the issue of mask-wearing was recognized to be part of daily life. The topics of lockdown (T4) and local businesses (T8) consumed a large proportion in the sequence, owing to the problem of mask-wearing at local businesses. Furthermore, the spread of infectious diseases was strongly correlated to large gatherings of people. Accordingly, the topic of mandatory mask-wearing (T1) gradually gained consensus and showed a trend of international compliance. Anticipation for clinical management (T10) later emerged, focusing on vaccinations. Meanwhile, topics on the economic crisis (T6) and protests (T5) were emphasized. Issues of political accountability then emerged. Additionally, a strong mask-wearing refusal emerged as a personal freedom issue. Quarantine management (T13) issues related to COVID-19 were also identified. Because the number of confirmed cases in Korea increased at the beginning of the outbreak, we noticed that neighboring countries blocked access to Korea. However, this negative position changed as the number of confirmed cases in Korea was recognized as a clear indicator of causation. Topics related to the US presidential election (T3), public sports events (T11), Covid-19 outbreaks in China (T9), and related educational issues (T2) appeared.

This study reports inter-topic connectivity and shows that quarantine management (T13) was a network hub for topic discussion for (T13) mediating movement restrictions (T4), additional outbreaks (T9), aviation curtailments (T7), and clinical management (T10). Outbreaks (T9), progress (T4), responses (T10, T13), and derivative industrial impacts (T7) were all connected.

In summary, the current study contrasted key topics before and after the pandemic declaration (RQ1) and demonstrated relationships among topics (RQ2). In the face of the COVID-19 crisis, future research may further provide discursive/ thematic analyses based on the findings of this research.

## 6. Conclusions

This study applied a big-data analysis methodology to analyze the topics of international media reports on the crisis surrounding the COVID-19 pandemic situation, focusing on the issue of mask-wearing. We applied an STM methodology to help understand how the weight of the topic changed over time, before and after the WHO pandemic declaration. Therefore, topic network analysis was performed by calculating the degree of similarity among mask-wearing topics.

Our study reconfirmed the statistical significance of big-data analysis in social science fields. Through this, the differences between the topics emphasized before and after the pandemic declaration were analyzed.

This research was not limited to media reports in a specific country; instead, it integrated a multicultural perspective. The analysis of the differences among topics according to specific nationalities and cultural regions is possible using this approach. Furthermore, it is unique because it merges social and data sciences with big data analysis, in contrast to the existing approach of content analysis.

As a follow-up to this study, it will be useful to examine the differences between topics from specific countries or cultural regions. Furthermore, it will be possible to analyze various trends in issues by augmenting news articles published in 2021 and beyond.

## Figures and Tables

**Table 1:** Top-20 news media by press volume.

Newspaper	Count	Cluster
The New York Times	4794	North America
National Post (f/k/a The Financial Post) (Canada)	3920	North America
The Independent (United Kingdom)	3278	Europe
Agence France Presse (English)	2867	Europe
Xinhua General News Service	2712	Asia
mirror.co.uk	2584	Europe
The Guardian (London)	2197	Europe
The Straits Times (Singapore)	1522	Asia
The Sun (England)	1392	Europe
The Daily Telegraph (London)	1328	Europe
The Times (London)	1283	Europe
The Toronto Star	1281	North America
South China Morning Post.com	1168	Asia
dpa international (Englischer Dienst)	1151	Europe
The Philadelphia Inquirer	1074	North America
China Daily	1054	Asia
China Daily (Hong Kong Edition)	1030	Asia
Daily Mirror	1014	Europe
The New Zealand Herald	984	Europe
The Globe and Mail (Canada)	811	North America

**Table 2:** Parameters of STM.

STM Parameters	
Categorical variables	Asia, Europe, North America
Time-series variables	Day
Number of topics	13
lower.thresh	1000
Random seed	2020

**Table 3:** Topic label and keywords.

Topic	Words
T1—Mandatory wearing masks	FREX: wearing_masks, mask, covering, face, wear, mandatory, fine
T2—Education	FREX: school, student, teacher, class, parent, child, education
T3—President election	FREX: biden, trump, white_house, election, republican, president, voter
T4—Lockdown	FREX: restriction, lockdown, death, case, record, rise, france
T5—Protesting movement	FREX: protester, police, protest, arrest, violence, fire, demonstration
T6—Economic crisis	FREX: economy, crisis, johnson, leader, job, labor, ballot
T7—Aviation industry	FREX: passenger, flight, airline, airport, travel, plane, crew
T8—Local business	FREX: restaurant, customer, store, business, employee, park, reopen
T9—Outbreak of COVID-19 in China	FREX: chinese, wuhan, china, beijing, campus, epidemic, shanghai
T10—Clinical management	FREX: doctor, vaccine, hospital, nurse, care, patient, flu
T11—Sport game	FREX: player, game, fan, league, season, football, match
T12—Life and family	FREX: love, film, mother, father, friend, story, husband
T13—Quarantine management	FREX: korea, contact, japan, quarantine, confirm, facility, test

**Table 4:** Estimated effect of STM.

Topic	Coefficients:	Estimate	Std. Error	*t* Value	Pr(>|t|)
T1—Mandatory wearing masks	covidbefore	−0.002528	0.005071	−0.499	0.618028
T2—Education	covidbefore	−0.0080632	0.0040691	−1.982	0.04753 *
T3—President election	covidbefore	−0.009996	0.005965	−1.676	0.093798
T4—Lockdown	covidbefore	−0.007482	0.006965	−1.074	0.282756
T5—Protesting movement	covidbefore	−0.12609	0.00676	−18.653	<2 × 10^−16^ ***
T6—Economic crisis	covidbefore	−0.026295	0.004992	−5.267	1.39 × 10^−7^ ***
T7—Aviation industry	covidbefore	0.019040	0.004162	4.575	4.78 × 10^−6^ ***
T8—Local business	covidbefore	−0.009174	0.005636	−1.628	0.10362
T9—Outbreak of COVID-19 in China	covidbefore	0.073914	0.006367	11.608	<2 × 10^−16^ ***
T10—Clinical management	covidbefore	0.0148984	0.0064882	2.296	0.02167 *
T11—Sport game	covidbefore	0.012398	0.005396	2.298	0.0216 *
T12—Life and family	covidbefore	−0.0008624	0.0080104	−0.108	0.914268
T13—Quarantine management	covidbefore	0.070270	0.005733	12.256	<2 × 10^−16^ ***

Note: *: *p* < 0.05; ***: *p* < 0.001. Coefficients are calculated from “covidbefore” which means if the estimates are >0 (+), then the topic is close to “before WHO pandemic declaration”. If the estimates are <0 (−), then it is close to “after WHO pandemic declaration”.
